# Investigation of the Effect of Local Melatonin Application on Biomechanic Bone-Implant Connection in Titanium Implants with Different Surface Placed in Rat Tibias

**DOI:** 10.3390/medicina62091691

**Published:** 2026-09-03

**Authors:** Kevser Sökmen, Serkan Dündar, Erhan Cahit Ozcan, Murat Tanrısever, Ozmen Istek, Umit Koray Can

**Affiliations:** 1 Department of Periodontology, Faculty of Dentistry, Alanya Alaaddin Keykubat University, Alanya 07400, Antalya, Turkey; 2 Department of Periodontology, Faculty of Dentistry, Fırat University, Elazığ 23119, Turkey; sdundar@firat.edu.tr; 3 Department of Plastic, Esthetic and Reconstructive Surgery, Faculty of Medicine, Fırat University, Elazığ 23119, Turkey; ecozcan@firat.edu.tr; 4 Department of Surgery, Faculty of Veterinary Medicine, Fırat University, Elazığ 23119, Turkey; mtanrisever@firat.edu.tr; 5 Department of Nursing, Faculty of Health Sciences, Muş Alparslan University, Muş 49250, Turkey; o.istek@alparslan.edu.tr; 6 Turkish Jokey Club, Elazig Racecourse Horse Hospital, Elazığ 23350, Turkey; koraycan23@gmail.com

**Keywords:** bone implant connection, osseointegration, reverse torque analysis, biomechanic, local melatonin application

## Abstract

*Background and Objectives*: The aim of this study was to investigate the effects of local melatonin application on osseointegration of machined (MAC), resorbable blast material (RBM), and sandblasted and acid-etched (SLA) surface implants placed in rat tibiae, utilizing the reverse torque analysis. *Materials and Methods*: A total of 72 female Sprague-Dawley rats, weighing between 270 and 300 g, were included in the study. The rats were divided into six groups, and samples in which the implants were not properly placed were excluded from the study: control groups consisting of MAC-CNT (n = 12), RBM-CNT (n = 11), and SLA-CNT (n = 12), and local melatonin (MLT)-treated groups consisting of MAC-MLT (n = 12), RBM-MLT (n = 10), and SLA-MLT (n = 10). The implants were surgically placed into the tibiae of the rats under general anesthesia. Following a four-week experimental period, the biomechanical bone–implant connection level was evaluated using reverse torque analysis. *Results*: The lowest mean biomechanical bone–implant connection value was observed in the MAC-CNT group, whereas the highest value was recorded in the SLA-MLT group. Compared with the MAC-CNT group, all other groups demonstrated statistically significantly higher biomechanical connection values (*p* < 0.05). The SLA-MLT group showed significantly higher osseointegration levels than both the MAC-MLT and RBM-MLT groups (*p* < 0.05). In contrast, no significant difference was detected between the MAC-MLT and RBM-MLT groups (*p* > 0.05). Both MAC-MLT and SLA-MLT groups exhibited statistically significantly higher biomechanical bone-implant connection values compared to control groups (*p* < 0.05). *Conclusions*: Local melatonin application positively affected osseointegration in SLA and MAC surfaced implants. In contrast, local melatonin application did not provide any additional contribution to osseointegration in RBM surfaced implants.

## 1. Introduction

In contemporary dentistry, dental implants represent a highly predictable and widely utilized therapeutic approach for restoring missing dentition. The clinical predictability of these fixtures heavily relies on osseointegration, which is characterized as the direct structural and functional anchorage achieved between viable bone tissue and the implant’s biomaterial surface [1]. Successful osseointegration is achieved through the biological harmony between bone resorption mediated by osteoclastic activity and bone formation mediated by osteoblastic activity, a process referred to as bone remodeling [2]. Any factor affecting this dynamic process of bone healing and remodeling may lead to complications in the bone surrounding dental implants and even result in implant loss [3].

Parameters such as the surface topography of dental implants (micro- and nanoscale roughness), chemical composition, surface energy, and hydrophilic properties play a decisive role in successful osseointegration and the long-term stability of implants. Therefore, various surface modification methods have been developed to improve the biological response of implant surfaces. These methods include TiUnite anodization, resorbable blast media (RBM) blasting, acid etching, sandblasting and acid etching (SLA), NanoTite anodization, plasma spraying/titanium plasma spraying, laser ablation, and biological coatings such as hydroxyapatite coatings [4]. In addition, to enhance the osseointegration success of dental implants, growth factors and hormones such as estradiol, parathyroid hormone, and melatonin—which can directly influence bone healing—and anti-osteoclastic drugs are applied locally or systemically to the implant surface [5,6]. Recent studies have focused on the effects of these different biological agents on the osseointegration success of implants with varying surface characteristics [7,8,9].

Melatonin is a sleep hormone primarily produced by the pineal gland and plays a key role in regulating the circadian rhythm. In addition to its anti-inflammatory and antioxidant properties, melatonin exerts significant effects on bone metabolism. It suppresses osteoclastic activity by promoting the formation of type I collagen fibers [10]. Furthermore, it contributes to bone formation by stimulating the differentiation and proliferation of mesenchymal stem cells into osteoblasts [2,10]. Melatonin has also been shown to accelerate angiogenesis, the process of new blood vessel formation. This effect suggests that melatonin may provide additional support to the bone healing process, which is closely associated with vascularization [11]. Considering its effects on inflammatory responses and bone metabolism, melatonin has emerged as a promising molecule for implant applications. Numerous studies have demonstrated that local melatonin application promotes bone healing and positively affects the osseointegration of implants [12,13,14].

According to the literature, there is no study investigating the effect of melatonin, which is known to have beneficial effects on bone tissue, on the osseointegration of implants with different surface characteristics. Therefore, this experimental study aims to biomechanically evaluate the effect of local melatonin administration on bone–implant connection in titanium implants with different surface properties placed in rat tibiae, using a reverse torque analysis.

## 2. Materials and Methods

Ethical approval for all the experimental procedures and surgical protocols was officially granted by the Animal Experiments Local Ethics Committee of Fırat University (Approval No. 13–16, 6 August 2025). Every experimental phase of this study was conducted at the Experimental Research Center of Fırat University (Elazığ, Türkiye). Throughout the study period, all procedures involving animals were performed in strict accordance with the ARRIVE guidelines and the relevant regulations governing the use of experimental animals in research. All rats utilized in the investigation were sourced from the identical breeding institution, namely the Experimental Research Center of Fırat University (Elazığ, Türkiye).

### 2.1. Implant Surface Characterization

A total of 72 sterile endosseous titanium implants manufactured from Ti-6Al-4V alloy (Implance Dental Implant Systems, AGS Medical, Istanbul, Türkiye), with a diameter of 2.5 mm and a length of 4 mm, were used in the study. Three different implant surface topographies were evaluated: MAC, SLA, and RBM surfaces. Based on previously published literature, target surface roughness (Ra) ranges were specified for each implant surface prior to manufacturing. MAC implants were manufactured with a lower target Ra value than the SLA and RBM implants, whereas a target Ra range of 1–2 μm was specified for the SLA and RBM implants [15]. These specifications were communicated to the manufacturer, and the implants were custom manufactured accordingly. However, the Ra values of the specific implant lot used in this study were not independently measured or verified by the authors.

### 2.2. Animals and Study Design

A total of 72 female Sprague–Dawley rats, with an age range of 6 to 7 months, were utilized to perform this experimental investigation [16]. At baseline, the animals had body weights ranging from 270 to 300 g. The rats were housed in specially designed cages under controlled environmental conditions, including a temperature of 22 ± 2 °C and relative humidity of 55%, with a 12 h light/12 h dark cycle. Standard laboratory chow and water were provided ad libitum throughout the experimental period. To determine the appropriate animal cohort size before initiating the study, a sample size estimation was conducted through G*Power software (version 3.1.9.4; Franz Faul, Kiel University, Kiel, Germany). The power analysis indicated that to achieve a statistical power of 90% with an effect size of 0.5 and a significance threshold (α) set at 0.05 across the six experimental cohorts, a minimum sample size of eight rats per group was essential [17]. Although power analysis indicated that at least eight rats per group were required, twelve rats were included in each group to compensate for the risk of rat mortality and to increase the reliability of the findings. To compensate for potential mortality risks during the surgical interventions and the subsequent experimental follow-up, each group initially comprised 12 rats. To ensure hormonal standardization, vaginal smear examinations were performed, and all animals were confirmed to be in the same stage of the estrous cycle. Seventy-two female rats were randomly assigned to six equal groups using a computer-generated randomization protocol. Animals in which proper implant placement could not be achieved were excluded from the study.

- Machined Surface Control Group (MAC-CNT) (n = 12): Machined-surface titanium implants were inserted into the right tibiae of the animals. Following a 4-week healing interval, the animals were euthanized for subsequent biomechanical evaluation.
- RBM Surface Control Group (RBM-CNT) (n = 12): RBM-surface titanium implants were inserted into the right tibiae of the animals. Following a 4-week healing interval, the animals were euthanized for subsequent biomechanical evaluation.
- SLA Surface Control Group (SLA-CNT) (n = 12): SLA-surface titanium implants were inserted into the right tibiae of the animals. Following a 4-week healing interval, the animals were euthanized for subsequent biomechanical evaluation.
- Machined Surface Local Melatonin Group (MAC-MLT) (n = 12): Following local administration of 3 mg of powdered melatonin into the implant osteotomy site to the maximum capacity of the prepared implant bed, machined-surface titanium implants were inserted into the right tibiae of the animals. Following a 4-week healing interval, the animals were euthanized for subsequent biomechanical evaluation [16].
- RBM Surface Local Melatonin Group (RBM-MLT) (n = 12): Following local administration of 3 mg of powdered melatonin into the implant osteotomy site to the maximum capacity of the prepared implant bed, RBM-surface titanium implants were inserted into the right tibiae of the animals. Following a 4-week healing interval, the animals were euthanized for subsequent biomechanical evaluation [16].
- SLA Surface Local Melatonin Group (SLA-MLT) (n = 12): Following local administration of 3 mg of powdered melatonin into the implant osteotomy site to the maximum capacity of the prepared implant bed, SLA-surface titanium implants were inserted into the right tibiae of the animals. Following a 4-week healing interval, the animals were euthanized for subsequent biomechanical evaluation [16].

### 2.3. Surgical Procedures

**Figure 1:**
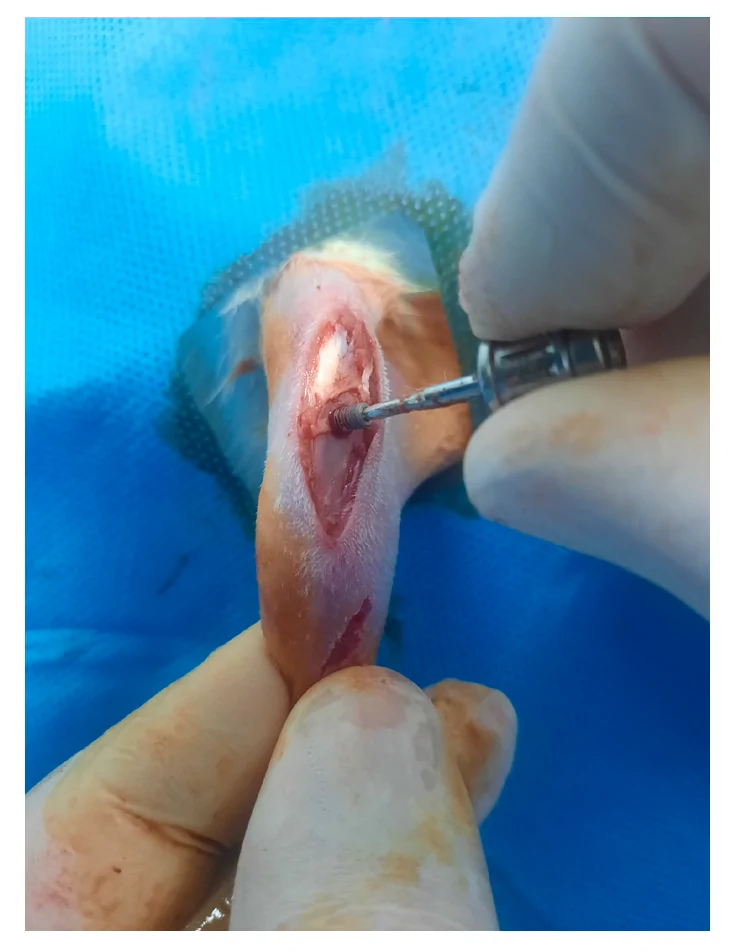
Surgical placement of titanium implants measuring 4 mm in length and 2.5 mm in diameter into the cortico-cancellous bone of the right tibiae of the rats.

All surgical procedures were performed under general anesthesia under sterile conditions. General anesthesia was induced by intraperitoneal administration of xylazine (5 mg/kg) and ketamine (50 mg/kg) using an appropriate syringe. The surgical site was shaved and disinfected with povidone-iodine solution. To access the metaphyseal region of the right tibia for implant placement, a 2 cm incision was created above the tibial crest using a No. 15 scalpel. During the preparation of the implant osteotomy sites, sterile saline solution was continuously used for irrigation to prevent thermal bone injury. Titanium implants (Ti-6Al-4V; Implant Dental Implant Systems, AGS Medical, Istanbul, Türkiye), measuring 4 mm in length and 2.5 mm in diameter, were inserted into the cortico-cancellous bone of the right tibiae of the rats with a standardized torque of 25 N/cm. The soft tissues were closed using 4–0 resorbable sutures (polyglactin) (Figure 1). Following surgery, antibiotic (penicillin, 50 mg/kg) and analgesic (tramadol hydrochloride, 0.1 mg/kg) treatments were administered intramuscularly for 3 consecutive days to prevent postoperative infection and to manage pain.

### 2.4. Biomechanical Analysis

**Figure 2:**
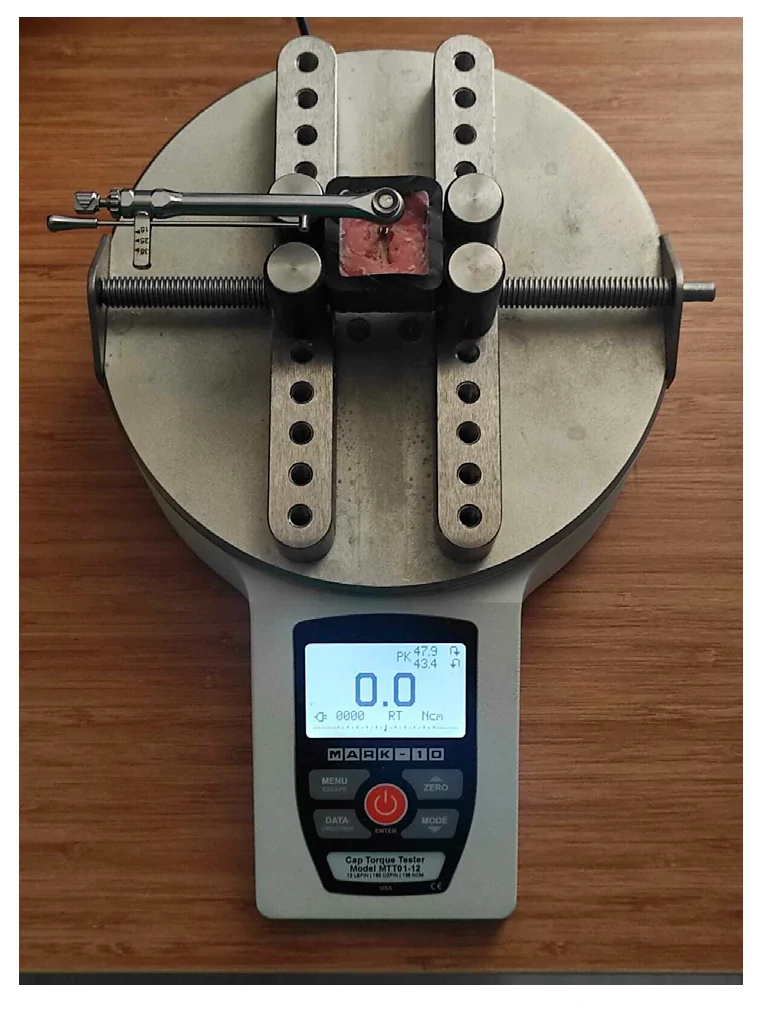
Performing the reverse torque analysis with a biomechanical testing machine (Mark-10 Corporation, Copiague, NY, USA).

Upon completion of the 4-week post-operative healing phase, euthanasia was performed on the animals. Subsequently, the titanium fixtures, along with the adjacent osseous structures, were meticulously harvested and immersed in a formaldehyde solution for preservation. The degree of osseointegration was evaluated using a reverse torque analysis performed with a biomechanical testing device (Mark-10 Corporation, Copiague, NY, USA) by a blinded examiner (M.T.) who was unaware of the group allocations. The force required to initiate the first rotation of the implant within the socket was recorded as the biomechanical osseointegration value. A hexagonal driver was engaged into the internal hex connection of the implant, and force was applied using a ratchet system. Care was taken to maintain a 90° angle between the ratchet and the hex driver during torque application. All measurements were recorded in Newton/centimeters (N/cm) (Figure 2).

### 2.5. Statistical Analysis

Statistical analyses were performed using IBM SPSS Statistics software (version 22.0; IBM Corp., Armonk, NY, USA). The normality of the data distribution was assessed using the Shapiro–Wilk test, and homogeneity of variances was evaluated with Levene’s test. Levene’s test was significant (F(5,61) = 6.115, *p* < 0.001), indicating that the homogeneity-of-variance assumption was violated. To address this heterogeneity while preserving the 2 × 3 factorial structure, the final analysis used SPSS GENLIN with a normal distribution, identity link, and robust covariance estimator. Main and interaction effects were evaluated with Type III Wald chi-square tests. After the significant KM × Groups interaction, simple effects were evaluated from estimated marginal means with Bonferroni adjustment. *p*-value of <0.05 was considered statistically significant.

## 3. Results

Throughout the experimental period, no mortality, postoperative infection, or wound dehiscence was observed among the animals. Due to improper implant placement, one specimen from the RBM-CNT group and two specimens each from the RBM-MLT and SLA-MLT groups were excluded from the final analysis.

Robust generalized linear model results for biomechanical bone-implant connection values are shown in Table 1. Levene’s test indicated significant heterogeneity of variances across the six experimental groups (F(5,61) = 6.115, *p* < 0.001). Therefore, biomechanical bone–implant connection values were analyzed using a generalized linear model with a normal distribution, identity link function, and robust covariance estimator. Type III Wald tests showed significant effects of melatonin application (Wald χ^2^(1) = 9.152, *p* = 0.002), implant surface (Wald χ^2^(2) = 69.268, *p* < 0.001), and melatonin application-by-surface interaction (Wald χ^2^(2) = 19.833, *p* < 0.001). Because the interaction was significant, simple-effect comparisons were examined using estimated marginal means.

Pairwise comparisons of implant surfaces under control and melatonin conditions are presented in Table 2. Within the control condition, the simple effect of implant surface was significant (Wald χ^2^(2) = 63.775, *p* < 0.001). Both RBM and SLA surfaces showed significantly higher biomechanical bone–implant connection values than MAC surface (both Bonferroni-adjusted *p* < 0.001), whereas RBM and SLA did not differ significantly (*p* = 1.000). Within the melatonin condition, the simple effect of implant surface was also significant (Wald χ^2^(2) = 18.135, *p* < 0.001). SLA showed significantly higher biomechanical bone–implant connection values than both MAC (*p* < 0.001) and RBM (*p* = 0.002), whereas the MAC-RBM difference was not significant (*p* = 0.096).

A comparison of control and melatonin conditions on each implant surface is presented in Table 3. Within-surface comparisons showed that local melatonin application was associated with significantly higher biomechanical bone–implant connection values for MAC implants (mean difference = 1.28 N/cm, Wald χ^2^(1) = 21.825, *p* < 0.001) and SLA implants (mean difference = 1.95 N/cm, Wald χ^2^(1) = 6.701, *p* = 0.010). In contrast, no statistically significant difference was observed between the control and melatonin groups for RBM implants (mean difference = −0.57 N/cm, Wald χ^2^(1) = 2.551, *p* = 0.110).

## 4. Discussion

Although numerous studies have investigated the effects of melatonin on the osseointegration of dental implants [13,18,19], no study has, to the best of our knowledge, evaluated this effect in the context of implants with different surface characteristics. Therefore, the present study aimed to investigate the effect of locally applied melatonin on bone–implant integration around titanium implants with three distinct surface characteristics (MAC, RBM, and SLA) placed in rat tibiae. The findings demonstrated that differences in implant surface modifications influenced the degree of osseointegration achieved. Furthermore, local melatonin application was found to enhance osseointegration in implants with both MAC and SLA surface characteristics.

In the present study, the robust factorial analysis demonstrated significant effects of both implant surface characteristics and local melatonin application on biomechanical bone–implant integration, with a significant interaction between these factors. The implant surface showed a significant effect on biomechanical bone–implant integration (Wald χ^2^(2) = 69.268, *p* < 0.001), while treatment condition also had a significant effect (Wald χ^2^(1) = 9.152, *p* = 0.002). Importantly, the significant interaction between melatonin application and implant surface (Wald χ^2^(2) = 19.833, *p* < 0.001) indicates that the effect of melatonin was not uniform across the different implant surfaces. Consistent with previous studies, this finding supports the important role of implant surface characteristics, including surface topography and wettability, in regulating protein adsorption, osteoblast attachment, and subsequent bone formation at the bone–implant interface, thereby influencing osseointegration [20,21,22]. Furthermore, the surface-dependent response to melatonin suggests that the effect of this biological stimulus may be modulated by the physicochemical properties of the implant surface. In the present study, local melatonin application significantly increased biomechanical bone–implant integration for Machined and SLA implants, whereas no significant difference was observed for RBM implants. Similarly, within the melatonin condition, SLA implants exhibited significantly higher biomechanical bone–implant integration than both Machined and RBM implants. These findings suggest that the response to melatonin may depend on the characteristics of the implant surface. Similar surface-dependent effects have been reported for other bioactive agents involved in bone metabolism, emphasizing the potential importance of interactions between biological stimuli and implant surface modifications during osseointegration [15,23,24].

Implant surface topography is widely recognized as one of the key determinants of successful osseointegration. Current evidence suggests that moderately rough implant surfaces provide a more favorable biological environment for osteoblast adhesion, proliferation, and subsequent bone mineralization. Lagdive et al. demonstrated that surfaces with moderate roughness (Ra ≈ 1.5 μm) optimize osteoblastic activity and bone formation [20], whereas other studies have reported that excessively rough surfaces may increase bacterial colonization without providing additional benefits for bone–implant integration compared with moderately rough surfaces [21]. Collectively, these findings indicate that the relationship between surface roughness and osseointegration is not simply linear; rather, there appears to be an optimal range of roughness that promotes a favorable biological response. In the present study, the superior biomechanical bone–implant connection observed in the RBM-CNT and SLA-CNT groups compared with the MAC-CNT group is consistent with this concept, as both RBM and SLA implants possess moderately rough surfaces, whereas MAC implants exhibit a comparatively smoother topography. These findings further support the concept that moderate surface roughness enhances implant stability by promoting a more favorable bone healing response.

Despite the well-documented advantages of moderately rough implant surfaces, the relative superiority of different surface modification techniques remains inconclusive. Bingül et al. [17] reported the lowest osseointegration levels for MAC surfaces and the highest levels for RBM surfaces, supporting the advantage of moderately rough implant surfaces. Similarly, several studies have demonstrated that both RBM and SLA surfaces promote greater osteoblastic activity and bone-to-implant contact than MAC surfaces [22,25]. In contrast, Özcan et al. [15] found that SLA implants exhibited greater biomechanical osseointegration than both RBM and MAC surfaced implants in the presence of simultaneous allogeneic bone grafting. These discrepancies suggest that implant surface characteristics alone may not fully determine osseointegration outcomes. Instead, factors such as the experimental model, bone quality, adjunctive grafting procedures, healing period, and assessment methods are also likely to influence the biological response. Therefore, comparisons between different implant surface modifications should be interpreted within the context of the specific experimental conditions rather than assuming the superiority of one surface treatment over another. Consistent with the overall trend reported in the literature, the present study demonstrated significantly higher biomechanical bone–implant connection values in both the RBM-CNT and SLA-CNT groups than in the MAC-CNT group, whereas no significant difference was observed between the RBM and SLA surfaces. These findings reinforce the advantage of moderately rough implant surfaces over machined surfaces while suggesting that different surface modification techniques producing comparable levels of surface roughness may not necessarily result in significantly different biomechanical performance.

Accumulating evidence suggests that the biological effects of locally administered melatonin on osseointegration are dose dependent. Experimental studies have shown that a 3 mg local dose enhances bone healing more effectively than lower doses, resulting in greater bone-to-implant contact and increased new bone formation around implants [16,26]. In contrast, studies using a lower dose of 1.2 mg have failed to demonstrate significant improvements in bone-to-implant contact or bone density compared with untreated controls during the healing period [27]. These findings collectively indicate that the osteogenic potential of melatonin may become more evident only above a certain therapeutic threshold. Based on this evidence, a 3 mg dose of locally administered melatonin was selected in the present study to maximize its potential biological effects on peri-implant bone healing. Consistent with the existing literature, local melatonin application enhanced osseointegration, with the greatest improvement observed in implants with SLA and, to a lesser extent, machined surface characteristics. These findings further support the concept that the beneficial effects of melatonin on osseointegration are influenced not only by dosage but also by the biological interaction between the bioactive agent and implant surface characteristics.

Although most evidence supports the positive effect of locally applied melatonin on peri-implant bone healing, the magnitude of this effect appears to vary according to the implant surface characteristics. Clinical studies have demonstrated that local melatonin administration is associated with favorable implant stability, reduced marginal bone resorption, and enhanced peri-implant bone formation around SLA-surfaced implants [28,29]. The present findings are consistent with these studies, as the SLA-MLT group exhibited the highest biomechanical bone–implant connection values among all melatonin-treated groups. Rather than suggesting that melatonin alone is responsible for this outcome, these results indicate that its osteogenic effects may be potentiated when combined with implant surfaces that already provide a favorable microenvironment for cellular attachment and bone remodeling. This interaction may explain why the greatest biomechanical benefit of melatonin was observed in the SLA implants.

The influence of locally administered bioactive agents on osseointegration has also been shown to depend on implant surface characteristics, regardless of the specific pharmacological compound used. Bingül et al. demonstrated that local administration of zoledronic acid resulted in superior biomechanical bone–implant interface strength in SLA and RBM implants compared with MAC implants, highlighting the importance of the interaction between surface topography and biological stimulation [17]. Similarly, the present study demonstrated significantly higher biomechanical bone-implant connection values in the SLA-MLT group compared to both the RBM-MLT and MAC-MLT groups, confirming the superior response of SLA surfaces after local application of melatonin, a bioactive agent. However, unlike the findings reported by Bingül et al., RBM implants did not exhibit significantly greater biomechanical performance than MAC implants after melatonin application. This difference suggests that the biological response to local pharmacological modulation may not be uniform across different implant surface modifications. Variations in the mechanisms of action of melatonin and zoledronic acid, together with differences in implant surface microtopography, implant systems, and experimental protocols, may have contributed to these divergent findings. Consequently, the effectiveness of locally applied osteogenic agents should be interpreted as the result of a complex interaction between the pharmacological properties of the agent and the physicochemical characteristics of the implant surface, rather than as an isolated treatment effect.

Clinical and experimental studies have shown that local melatonin administration improves peri-implant tissue healing by reducing marginal bone loss, probing depth, and gingival inflammation, while simultaneously enhancing osteoblastic activity, bone formation, and bone density [30,31]. Beyond its role in bone formation, melatonin has also been reported to modulate the inflammatory response by suppressing pro-inflammatory cytokines, promoting angiogenesis, inhibiting osteoclastogenesis, and stimulating osteogenesis, thereby contributing to the prevention and delay of the progression of peri-implantitis [32]. Furthermore, experimental evidence suggests that these regenerative effects may occur independently of dose within a specific therapeutic range [33]. These findings support the concept that melatonin enhances biomechanic bone-implant connection not only by stimulating bone formation but also through multiple complementary biological pathways. Consistent with this evidence, local melatonin application significantly improved the biomechanical bone-implant connection in both MAC and SLA implant groups compared to their respective controls, confirming that melatonin has a positive effect on osseointegration under appropriate surface conditions.

One of the most interesting findings of the present study was the absence of a significant additional benefit of local melatonin application in the RBM implants. Park et al. [23] demonstrated that laser ablation of RBM implants did not further improve bone-to-implant contact compared with untreated RBM surfaces, indicating that additional surface modification may provide limited benefit once a biologically favorable surface has already been established. Similarly, Conserva et al. [24] reported that FasL expression, a molecule involved in bone preservation and osseointegration, was increased only on MAC surfaces, while RBM and Ca^2+^ nanostructured surfaces did not show further improvement since their microporous architectures already support early cell adhesion, fibrin retention and osteoblastic differentiation. The current findings, considered together with previous literature, suggest that the biological effects of melatonin are surface dependent and that the optimized microtopography of RBM implants may already provide conditions approaching maximal osteoblastic activity and new bone formation. Under such circumstances, the potential anabolic effects of melatonin may become less apparent because of a ceiling effect.

The present findings raise the possibility that the biological effects of locally applied melatonin are influenced not only by implant surface characteristics and surface roughness but also by additional biological and physicochemical factors. Lee et al. evaluated the effects of implants coated with a nano/micro-combined hydroxyapatite (HA) structure using a laser-induced one-step coating technique and reported that the surface roughness ranking was HA > RBM > SLA > MAC, whereas the surface wettability ranking was RBM > HA > MAC > SLA [22]. In a study by Sun et al., it was demonstrated that as implant material hydrophilicity increases, both cell adhesion and bone-binding capacity also increase [34]. Therefore, the combination of favorable surface roughness and superior wettability may enable RBM implants to achieve a high baseline level of osseointegration, leaving limited potential for further enhancement following local melatonin application. This concept is consistent with the present findings, in which melatonin significantly increased biomechanical bone–implant connection in the MAC and SLA groups but failed to produce an additional benefit in RBM implants. Rather than indicating a lack of biological activity of melatonin, these findings suggest that its osteogenic effect is highly dependent on the interaction between the bioactive agent and the physicochemical properties of the implant surface. Consequently, the absence of a significant response in the RBM group may reflect a biological ceiling effect, whereby the intrinsic characteristics of the RBM surface already provide conditions approaching maximal osteogenic potential.

Sex is increasingly recognized as an important biological variable influencing bone metabolism and implant osseointegration [35]. Female Sprague–Dawley rats were selected in the present study because they represent one of the most widely used and well-characterized experimental models for bone healing and implant osseointegration research owing to their reproducible skeletal physiology and healing response [36]. Although male rats have frequently been preferred in implant studies to eliminate the potential influence of fluctuations in estrogen levels [37], accumulating evidence indicates that bone remodeling is regulated not only by estrogen but also by androgen signaling through distinct molecular pathways [38,39]. Moreover, the influence of sex on implant osseointegration and implant survival remains controversial. While some clinical studies have reported higher implant failure rates in males [40,41], others have found no significant association between sex and implant outcomes [42]. Therefore, the use of a single sex in experimental studies is considered an appropriate strategy to reduce biological variability associated with sex-related hormonal differences and to improve experimental standardization [16]. In the present study, this standardization was further strengthened by synchronizing the estrous cycle of all female rats using vaginal smear analysis before surgery.

Melatonin has been shown to interact with sex hormone signaling and bone metabolism through multiple molecular pathways [43]; suggesting that its osteogenic effects may be influenced by the hormonal milieu. However, the current evidence is insufficient to conclude that the effects of melatonin on implant osseointegration or the biological response to different implant surface characteristics differ significantly between male and female animals. Therefore, although the present findings demonstrate that local melatonin application significantly enhances biomechanical osseointegration in female rats, particularly around machined and SLA-surfaced implants, caution should be exercised when extrapolating these results to males. Future studies directly comparing male and female animal models are warranted to clarify whether sex influences the interaction between implant surface characteristics, local melatonin application, and osseointegration.

In the present study, the effects of locally applied melatonin on osseointegration of titanium implants with different surface characteristics were evaluated using a rat tibia model. The lack of clinical-level assessments and the absence of systemic melatonin application represent the main limitations of this study. In addition, osseointegration was assessed using reverse torque analysis. Another limitation of the present study is the absence of histomorphometric, micro-CT, and immunohistochemical analyses.

## 5. Conclusions

Within the limitations of the present study, local melatonin application during implant surgery appears to positively influence osseointegration in SLA- and MAC-surfaced implants. However, local melatonin administration did not provide any additional benefit for bone integration in RBM-surfaced implants. Nevertheless, further well-designed long-term experimental and clinical studies with larger sample sizes, different dosages, and alternative administration routes (local or systemic) are required to more clearly elucidate the effects of melatonin on osseointegration across different implant surface characteristics.

## Figures and Tables

**Table 1:** Robust generalized linear model results for biomechanical bone-implant connection values.

Source of Variation	Wald χ^2^	df	*p*-Value
Melatonin application	9.152	1	0.002 *
Implant surface (Groups)	69.268	2	<0.001 *
Melatonin application × Implant surface	19.833	2	<0.001 *

Biomechanical bone–implant connection was analyzed using a generalized linear model with a normal distribution, identity link, Type III Wald tests, and a robust covariance estimator. Melatonin application consisted of control and local melatonin application groups. Implant surface consisted of MAC, RBM, and SLA surfaces. The analysis included 67 cases with valid biomechanical bone–implant connection measurements. * (*p* < 0.05). MAC: Machined; RBM: Resorbable Blast Material; SLA: Sandblasted and Acid-Etched.

**Table 2:** Pairwise comparisons of implant surfaces within the control and melatonin conditions.

Melatonin Application	Comparison	First Group, Mean ± SD (n)	Second Group, Mean ± SD (n)	Mean Difference ^1^	Bonferroni-Adjusted *p* *
CNT	RBM − MAC	5.69 ± 1.12 (n = 11)	3.31 ± 0.69 (n = 12)	2.38	<0.001 *
CNT	SLA − MAC	5.55 ± 1.07 (n = 12)	3.31 ± 0.69 (n = 12)	2.24	<0.001 *
CNT	SLA − RBM	5.55 ± 1.07 (n = 12)	5.69 ± 1.12 (n = 11)	−0.14	1.000
MLT	RBM − MAC	5.12 ± 0.52 (n = 10)	4.58 ± 0.71 (n = 12)	0.54	0.096
MLT	SLA − MAC	7.50 ± 2.31 (n = 10)	4.58 ± 0.71 (n = 12)	2.92	<0.001 *
MLT	SLA − RBM	7.50 ± 2.31 (n = 10)	5.12 ± 0.52 (n = 10)	2.38	0.002 *

^1^ Mean difference was calculated as the mean of the first-listed group minus the mean of the second-listed group. Pairwise comparisons were derived from estimated marginal means of the same robust factorial model. Bonferroni correction was applied separately to the three implant-surface comparisons within each treatment condition. The overall simple effect of implant surface was significant in both the Control group (Wald χ^2^(2) = 63.775, *p* < 0.001) and the Melatonin group (Wald χ^2^(2) = 18.135, *p* < 0.001). * (*p* < 0.05). CNT: Control; MLT; Melatonin; MAC: Machined; RBM: Resorbable Blast Material; SLA: Sandblasted and Acid-Etched.

**Table 3:** Comparisons of control and melatonin conditions within each implant surface.

Implant Surface	CNT, Mean ± SD (n)	MLT, Mean ± SD (n)	Mean Difference ^1^	Bonferroni-Adjusted *p*
MAC	3.31 ± 0.69 (n = 12)	4.58 ± 0.71 (n = 12)	1.28	<0.001 *
RBM	5.69 ± 1.12 (n = 11)	5.12 ± 0.52 (n = 10)	−0.57	0.110
SLA	5.55 ± 1.07 (n = 12)	7.50 ± 2.31 (n = 10)	1.95	0.010 *

^1^ Mean difference was calculated as MLT − CNT. Comparisons were derived from estimated marginal means of the same robust factorial model. Because treatment comparison has two levels, each surface contains one CNT-MLT comparison. Bonferroni-adjusted *p*-values are reported as produced by SPSS GENLIN. * (*p* < 0.05). CNT: Control; MLT: Melatonin; MAC: Machined; RBM: Resorbable Blast Material; SLA: Sandblasted and Acid-Etched.

## Data Availability

The raw data supporting the conclusions of this article will be made available by the authors on request.

## Abbreviations

MAC	Machined
RBM	Resorbable blast material
SLA	Sandblasted and acid-etched
CNT	Control
MLT	Melatonin
HA	Hydroxyapatite
